# Amulet™ Shines and Protects; Pacing Battle Intensifies with “More Leads or No Leads”?

**DOI:** 10.19102/icrm.2022.130110

**Published:** 2022-01-15

**Authors:** Christopher R. Ellis, Nicholas King

**Affiliations:** ^1^Vanderbilt Heart and Vascular Institute, Nashville, TN, USA

**Keywords:** Atrial fibrillation, cardiac implantable electronic devices, cardiac resynchronization therapy, leadless pacing

## Introduction

Arrhythmia device developments in 2021 provided long-awaited alternatives for stroke prevention in atrial fibrillation (AF) and both non-invasive and transcatheter enhancements to cardiac pacing. In brief, the Amulet™ device (Abbott, Chicago, IL, USA) was shown to be effective in preventing thromboembolism for patients in AF. Implantable loop recorders (ILRs) have shown that AF is highly prevalent even in the non-embolic stroke population. The Micra™ leadless pacemaker (Medtronic, Minneapolis, MN, USA) was upgraded to enhance atrioventricular (AV) synchrony. Finally, cardiac resynchronization therapy (CRT) is being enhanced by the addition of pacing sites in multi-site pacing or altering the lead placement to activate the native conduction system in left bundle branch area pacing (LBBAP).

We will review these developments, comment on future directions, and consider where these trials may have impacted current arrhythmia management in 2021.

## Device updates in atrial fibrillation

AF is the most common arrhythmia, and its true prevalence remains underestimated due to the large number of asymptomatic patients.^1^ Carrying substantial morbidity and mortality from cardioembolic stroke, advances have allowed for durable rhythm control with catheter-based ablation, improved detection with expanding options for ILR monitoring, and non-pharmacologic alternatives to anticoagulation.^2,3^ The potential impact on reducing the overall health care burden of disabling or fatal AF-related stroke cannot be overlooked.

### Atrial fibrillation stroke risk and left atrial appendage closure

AF confers an increased risk of embolic events stratified by the CHA_2_DS_2_-VASc score, which informs consideration of risk-modifying therapy.^3,4^ Previously, only vitamin K antagonists (VKAs) were available to reduce the risk of embolic events; however, the more recently developed novel or more appropriately named direct oral anticoagulants beginning with dabigatran in 2010 have been shown to be non-inferior to VKAs and are indicated for the prevention of embolic events.^3–5^ Given the risks of long-term systemic anticoagulation, non-pharmacologic management of AF’s embolic risk has been investigated by the closure of the left atrial appendage (LAA). The first percutaneously inserted intracardiac LAA occlusion (LAAO) device brought to market was the Percutaneous Left Atrial Appendage Transcatheter Occlusion device in 2001; however, it was subsequently withdrawn in 2006.^6^ The Lariat^®^ device (SentreHEART, Redwood, CA, USA) for epicardial closure of the LAA was approved in 2009 as a soft-tissue closure device but has not seen significant use in the United States (US).^7^

The Watchman™ LAAO device (Boston Scientific, Marlborough, MA, USA) was shown to be non-inferior to VKAs in the Watchman™ Left Atrial Appendage Closure Technology for Embolic Protection in Patients with Atrial Fibrillation (PROTECT-AF) and Evaluation of the Watchman™ Left Atrial Appendage Closure Device in Patients with Atrial Fibrillation vs. Long-term Warfarin Therapy (PREVAIL) trials, the US Food and Drug Administration subsequently approved it in 2015 as an alternative for oral anticoagulation.^8,9^ The Watchman™ device is currently in its second-generation iteration as the Watchman™ FLX was shown to be safer to implant and provide higher complete seal rates with lower device-related thrombus (DRT) than its predecessor.^10^ Surgical closure of the LAA was long thought to be protective against embolic events, and the landmark trial Left Atrial Appendage Occlusion Study III (LAAOS III) confirmed this, though 75% of patients were continued on oral anticoagulation.^11^ Other minimally invasive LAAO devices are under investigation in the US to enhance patient–device match (WaveCrest by Coherex Medical, Salt Lake City, UT, USA; Conformal LAA seal from Conformal Medical, Nashua, NH, USA).

This year, the results of the Amplatzer™ Amulet™ Left Atrial Appendage Occluder IDE Trial (IDE trial) against the first-generation Watchman™ 2.5 device were published in *Circulation*. The Amulet™ device is a self-expanding nitinol mesh lobe that is inserted into the LAA using 6–10 stabilizing wires and is connected to a polyester-covered disk that seals the LAA ostium. It is available in 8 different sizes with a minimum LAA depth of 12 mm.^6,12^ In comparison, the Watchman™ device is a self-expanding nitinol frame semi-covered by a polyethylene terephthalate membrane fabric held in by 10 anchors, while the Watchman™ FLX is fully covered by the membrane fabric with 12 anchors.^6,12^ The Amulet IDE trial randomized 1,878 patients in an intention-to-treat analysis to either the Watchman™ 2.5 device or Amulet™ device. The Amulet™ device was shown to be as effective as Watchman™ in the composite outcome of stroke, systemic embolism, or cardiovascular/unexplained death (5.6% vs. 7.7%; difference, −2.12; 95% confidence interval, −4.45 to 0.21; *P* < .001 for noninferiority). The procedural complications were higher in the Amulet™ group (4.5% vs. 2.5%) and more frequent with early case numbers consistent with an operator learning curve. The overall safety of the 2 devices was otherwise similar.^12^

With its 2-part construction and dual-seal mechanism, the Amulet™ device hopes to produce a better seal and therefore total LAAO. A well-seated device with the absence of an LAAO flow leak of 5 mm or greater has been shown in the long-term follow-up of the PROTECT-AF participants to be as protective as VKAs for stroke, embolism, and death.^9^ The Amulet IDE trial defined technical device success as a peri-device jet of less than 5 mm, which occurred in 96% of Amulet™ cases and 94.5% of Watchman™ cases, with a complete occlusion rate of 63% for the Amulet™ occluder and 46.1% for the Watchman™ device.^12^ The Protection Against Embolism for Nonvalvular Atrial Fibrillation Patients: Investigational Device Evaluation of the Watchman™ FLX Left Atrial Appendage Closure Technology (PINNACLE-FLX) trial of the Watchman™ FLX device similarly showed a peri-device leak of less than 5 mm in 100% of patients, with 7.4% having a residual leak between 0 and 5 mm.^10^ It has, however, been shown that a residual leak of even 3 mm or beyond confers an increased risk of embolic events, and more study is needed to determine if the Amulet™ does provide a better seal.^13^

**Figure 1:**
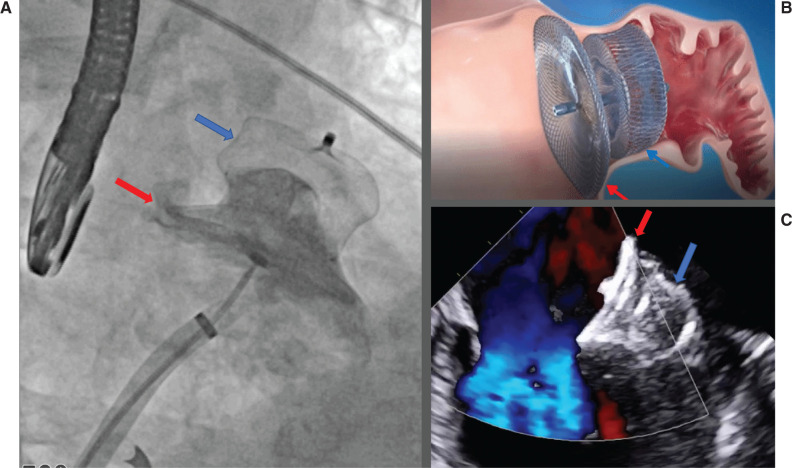
Amplatzer™ Amulet™ left atrial appendage (LAA) occlusion device. Blue arrows denote lobe attachment and the site of anchoring. Red arrows denote Amulet™ disc and position at the pulmonary vein ridge to the LAA ostium. **A:** A 34-mm device with angiogram showing occlusion of the LAA. **B:** Schematic of the target location. **C:** Transesophageal echocardiography showing chronic seal with no leak or DRT at 1-year device-related thrombus.

The increased rate of complete occlusion at the time of the procedure allowed more patients who received the Amulet™ occluder to be discharged off anticoagulation; however, at 9 months of follow-up, most patients from both groups were off anticoagulation.^12^ While no differences in non–procedure-related major bleeding were reported, the higher rate of complete immediate closure might be of clinical importance in patients who require rapid de-escalation from full anticoagulation. The novel design of the Amulet™ device also allows clinicians to better select a device to meet the anatomic needs of their patient’s LAA. It can accommodate shallower LAAs that would otherwise have deferred Watchman™ style devices and placed the patient on anticoagulation. Additionally, coverage of the large proximal posterior lobes and pits on the pulmonary vein–LAA ridge is more likely with Amulet™. Pericardial effusion rates in the first 30 days post-LAAO with Amulet™ were balanced by lower DRT rates despite a dual antiplatelet therapy-only post-implant regimen in the majority of subjects. Direct comparisons of Watchman™ FLX to Amulet™ as regards complete LAA closure by computed tomography angiography versus transesophageal echocardiography support a comparable occlusion rate. Real-world evidence of Amulet™ use in the US following commercial launch is needed as operator experience ramps up **(Figure 1)**.

### Detection of atrial fibrillation post-stroke

The incidence and duration of AF and its role in cardioembolic strokes have been one of intense investigation. The TRENDS study, which was an observational study of patients receiving a pacemaker or defibrillator device with an atrial lead, found an incidence of any atrial arrhythmia of 47% over a mean follow-up of 1.4 years, with an increased risk of thromboembolic events with increased duration (>5.5 hours) of atrial arrhythmia.^14^ The Asymptomatic Atrial Fibrillation and Stroke Evaluation in Pacemaker Patients and the Atrial Fibrillation Reduction Atrial Pacing Trial (ASSERT) also included a cohort of patients with atrial chamber leads and detected atrial tachyarrhythmias of more than 6 minutes in 10.1% of patients over a 3-month monitoring period. When followed for a mean of 2.5 years, those patients with a subclinical atrial tachyarrhythmia that was detected in the monitoring period were found to have an increased rate of ischemic strokes and systemic embolisms.^15^ Screening and Optimising Stroke Prevention in Atrial Fibrillation (SOS-AF) study of implanted devices identified 43% of patients with at least five minutes of AF over a mean follow-up of 2 years and correlated even five minutes of AF with an increased stroke risk.^16^ The Registry of Atrial Tachycardia and Atrial Fibrillation Episodes (RATE) prospective cohort of St. Jude devices found a 50% incidence of AF defined as ≥3 ectopic atrial beats, with an episode length of 20 seconds or more being associated with an increased risk of stroke.^17^ All of these studies are potentially confounded by the highly selected study populations that at baseline had elevated CHADS_2_ scores (TRENDS: mean, 2.2 points; ASSERT: means, 2.2 and 2.3 points; SOS-AF: 59% of patients ≥ 2 points; RATE: mean, 1.8 points).^14–17^

Recent studies have taken advantage of the improvements in safety, longevity, and clinical use of ILRs to better clarify the relationship of AF and stroke. The Cryptogenic Stroke and Underlying Atrial Fibrillation (CRYSTAL-AF) trial randomized patients with cryptogenic strokes to ILR versus usual care and reported an AF incidence of 8.9% in patients with ILRs versus 1.4% found by usual care.^18^ Both cohorts also had elevated mean CHADS_2_ scores of 3 points in the ILR arm and 2.9 points in the usual care arm.^18^ The Post-embolic Rhythm Detection with Implantable Versus External Monitoring (PER DIEM) trial also randomized patients with a median CHA_2_DS_2_-VASc score of 4 points to ILR or 4 weeks of external cardiac monitoring and found more than 2 minutes of AF in 15.3% of the ILR arm and 4.7% of the external monitor arm.^19^ This led to guidelines recommending long-term cardiac monitoring following a cryptogenic stroke.^4^

The STROKE-AF trial published this year looked to determine if the incidence of AF was different in patients with small and large vessel strokes. A total of 492 patients with a mean CHA_2_DS_2_-VASc score of five points were randomized in the acute post-stroke period to an ILR or usual care. AF of longer than 30 seconds was detected in 12.1% of patients with an ILR over the course of 12 months compared to 1.8% of patients receiving usual care, with most events occurring beyond 30 days post-stroke. Post-hoc analysis showed that 67% of patients with detected AF were started on anticoagulation and that placement of an implantable cardiac monitor was associated with a non-significant reduction in recurrent ischemic or hemorrhagic stroke.^20^

Further work is still needed to elucidate the pathophysiologic role of AF and stroke. AF is clearly associated with an increased risk of stroke; however, every study has been confounded by the shared risk factors of the CHA_2_DS_2_-VASc score that predispose to both stroke and AF. The CRYSTAL-AF and PER DIEM trials sought to establish the role of extended cardiac monitoring for AF in the setting of cryptogenic stroke; however, STROKE-AF showed that, even in non-embolic small vessel strokes, the incidence of AF was elevated. ILRs are effective at detecting AF; the question remains as to who needs to have AF detected.

Future studies need to look at the prospective use of ILRs to detect AF in populations with elevated CHA_2_DS_2_-VASc scores but without the clinical events of AF or stroke to see if early detection and treatment modify outcomes. The present evidence, and especially the similarity of AF detection rates between CRYSTAL-AF and STROKE-AF, remains confounded by the similarity of stroke and AF risk factors and calls into question the limited use of ILRs in the stroke population.

## Updates in cardiac pacing

Since the implantation of the first myocardial electrodes connected to a pulse generator in 1958, implantable cardiac pacemakers have undergone numerous innovations culminating in the multifunctional devices in use today.^21^

### Leadless ventricular pacemakers and atrioventricular synchrony

**Figure 2:**
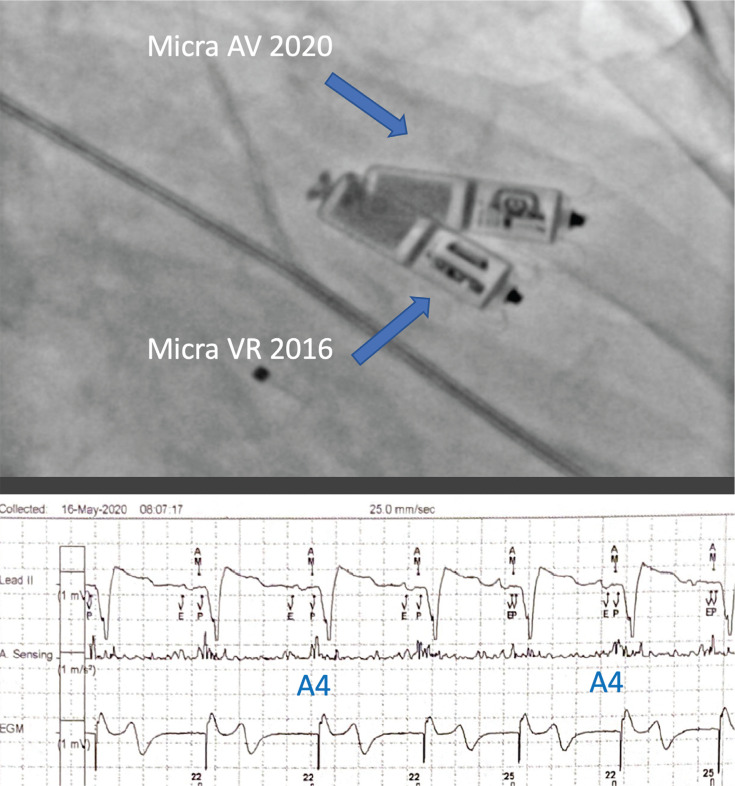
Patient with complete atrioventricular block 4 years after Micra™ VR implantation. Lower panel shows A4 sensing with atrial kick tracking 1:1 by device as the atrial mechanical marker for atrial mechanical signal, followed by ventricular pacing.

The Micra™ single-chamber pacemaker (Medtronic, Minneapolis, MN, USA) was introduced in 2016 as an alternative to single-lead pacemakers for VVI pacing.^22^ It has continued to function well and has been shown to have reduced rates of complications compared to transvenous pacemakers.^23,24^ However, its use is limited as a single-chamber pacing system as it lost the benefits of AV synchrony. A downloadable algorithm was designed and implemented in a prospective cohort with complete AV block to assess if AV synchrony could be added to device functionality in the Micra™ Atrial Tracking Using a Ventricular Accelerometer (MARVEL)-2 trial this year. The algorithm made use of the Micra™ device’s accelerometer to detect atrial filling and atrial kick to better time the ventricular stimulus. Following the download, the percentage of patients with synchronous AV pacing measured by surface Holter monitors increased from 26.8% to 89.2%. This translated to an increase in left ventricular (LV) stroke volume measured on echo.^25^ This represents an important proof of principle that a leadless ventricular pacemaker can achieve significant AV synchrony. While persistent AV dyssynchrony is a clear alteration of normal cardiac hemodynamics, trials are mixed as to whether there is a difference in outcomes apart from pacemaker syndrome with single-lead pacing versus dual-chamber pacemakers.^26^ To date, there is no clear minimum threshold of AV pacing to avoid the development of pacemaker syndrome; however, it is possible that some minimum amount of synchrony might avoid it even if not 100% synchronous. The development of Micra™ AV devices that restore a high percentage of AV synchrony might prevent the development of pacemaker syndrome, adding another advantage to the Micra™ or Micra™ AV system over traditional single-lead pacemakers. See **Figure 2**.

### Advances in cardiac resynchronization therapy

CRT uses an atrial lead and 2 ventricular leads (right ventricle [RV] and coronary sinus [CS]) to recreate the normal cardiac cycle by stimulating atrial and then biventricular (BiV) contraction. The Resynchronization Reverses Remodeling in Systolic Left Ventricular Dysfunction (REVERSE) trial, Multicenter Automatic Defibrillator Implantation Trial–Cardiac Resynchronization Therapy (MADIT-CRT), and Resynchronization–Defibrillation for Ambulatory Heart Failure Trial (RAFT) all showed benefit with CRT pacing in patients with reduced ejection fractions, symptoms of heart failure, and wide QRS complexes.^27–29^ The current recommendations suggest the use of BiV CRT if the QRS duration is more than 150 ms with a left bundle branch block morphology.^30^ However, some patients will not respond to CRT even in normal sinus rhythm with more than 95% paced beats, which has posed a clinical dilemma. To address this issue, 2 novel pacing schemes have been published this year: MSP and LBBAP.

The concept of MSP first began with the use of triple ventricular (TriV) site pacing, utilizing either 2 RV leads and 1 CS lead or 1 RV lead and 2 CS leads.^31–33^ Small trials showed mixed results of TriV pacing on symptoms but did consistently lead to improved echocardiographic function.^33–36^ Although promising, CRT using TriV pacing was limited by the era of devices it was investigated in, as devices only had 2 ventricular ports necessitating the use of a Y connector, which resulted in large current draws due to a drop in impedance.^37^ Quadripolar leads, first introduced in 2010 under the IS-4 connection standard to increase pacing configurations via “electronic repositioning,” allow for single-lead MSP. MSP via quadripolar leads has been shown to be safe and effective in improving LV function, but comparative effectiveness data have been lacking.^38–41^ The MultiPoint Pacing trial, a 6-month randomized trial of MSP via a quadripolar lead versus traditional BiV pacing, showed improved cardiac function and clinical outcomes with MSP.^42^ This year, the SMART-MSP trial randomized CRT non-responders to MSP or traditional BiV pacing and showed that 51% of non-responders had an improvement in a clinical composite score of mortality, heart failure events, New York Heart Association assessment, and a global symptom assessment with a minimal reduction in battery life.^43^ MSP via quadripolar leads is an exciting development in the use of CRT because it requires no change in technique for existing operators, the leads are already in use, and patients can realize an upgrade without any change in hardware.

LBBAP seeks to utilize the native conduction system to achieve more efficient ventricular stimulation. Prior work has shown that His-bundle pacing (HBP) was an effective strategy to achieve more efficient and synchronous ventricular capture.^44,45^ The Left Bundle Branch–Optimized Cardiac Resynchronization Therapy (LOT-CRT) trial was a feasibility study using a lead setup with a pacing CS lead, a defibrillation coil-only RV lead, and a pacing left bundle branch area (LBBA) lead in patients with a CRT indication. The LBBA lead was screwed across the septum approximately 1.5 to 2 cm distal to the His bundle to stimulate the left bundle branch. CRT utilizing the CS and LBBA leads in 91 patients resulted in a significantly narrower QRS complex compared to traditional BiV CRT pacing and LBBAP alone (means, 144, 181, and 170 ms, respectively). This corresponded to an increase in mean the LV ejection fraction from 28.5% to 37.2%, with additional improvements in the LV end-diastolic and end-systolic parameters.^46^ LBBAP holds several advantages over HBP, including indifference for the development of AF, which might be oversensed by HBP; preservation of lead ports for future implantable cardiac defibrillator upgrade; and early LV septal endocardial activation to bypass any potential disease of the conduction system.

CRT targeting more coordinated ventricular resynchronization using MSP and LBBAP seeks to improve outcomes for patients with heart failure. However, though it mechanistically fits that improvements in cardiac conduction will translate to better outcomes, further work is needed. Trials involving frontline use of MSP and LBBAP for CRT are needed to determine if the improvements in coordinated ventricular stimulation using these novel pacing techniques give an additional advantage over CRT or if simply achieving CRT is the deterministic outcome.

## Conclusions

This year has been another one marked by landmark developments in cardiac devices. The Amulet™ device offers immediate LAAO and is as effective as the Watchman™ device. ILRs are being increasingly deployed to unravel the link between AF and stroke. The Micra™ single-chamber leadless pacemaker was upgraded to have increased AV synchrony (Micra™ AV). Finally, CRT is being enhanced by adding additional pacing sites in MSP or altering the lead placement to activate the native conduction system in LBBAP. While further work is needed, these devices are already changing practice and will be coming increasingly into use as those additional trials are completed.
